# A Cocktail Method for Promoting Cardiomyocyte Differentiation from Bone Marrow-Derived Mesenchymal Stem Cells

**DOI:** 10.1155/2014/162024

**Published:** 2014-06-23

**Authors:** Qing Gao, Maojuan Guo, Xijuan Jiang, Xiantong Hu, Yijing Wang, Yingchang Fan

**Affiliations:** Key Laboratory of Pathology of State Administration of Traditional Chinese Medicine, School of Traditional Chinese Medicine, Tianjin University of Traditional Chinese Medicine, Tianjin 300193, China

## Abstract

A growing body of evidence supports the argument that bone marrow-derived mesenchymal stem cells (MSCs) can differentiate into cardiomyocyte-like cells in an appropriate cellular environment, but the differentiation rate is low. A cocktail method was designed: we investigated the role of 5-azacytidine (5-aza), salvianolic acid B (SalB), and cardiomyocyte lysis medium (CLM) in inducing MSCs to acquire the phenotypical characteristics of cardiomyocytes. The fourth-passage MSCs were treated with 5-aza, SalB, CLM, 5-aza+salB, 5-aza+CLM, SalB+CLM, and 5-aza+SalB+CLM for 2 weeks. Immunofluorescence results showed that cTnT expression in the 5-aza+salB+CLM group was stronger than other groups. Real-time qPCR and Western blotting analyses showed that cTnT, alpha-cardiac actin, mef-2c, Cx43, and GSK-3beta expression increased while beta-catenin expression decreased. The salB+5-aza+CLM group had the most evident effects. SalB combined with 5-aza and CLM improved cardiomyocyte differentiation from MSCs. In the MSCs differentiation process, the Wnt/beta-catenin signaling pathway had been inhibited.

## 1. Introduction

In 1999, Makino et al. first reported that bone marrow MSCs could differentiate into cardiomyocytes* in vitro* [1]. The use of 5-azacytidine (5-aza) is effective for cardiomyocyte differentiation, but it is clinically toxic [2]. Several other methods have been used to promote the differentiation of stem cells into cardiomyocytes, including the coculture of stem cells [3], treatment with cardiac tissue extracts [4], angiotensin II [5], and nitric oxide donors [6, 7]. Most of these methods are inefficient, generating only a small percentage of differentiated cells.

5-Aza, as a chemical analogue of cytidine, is generally known as a demethylation pharmaceutical that can induce MSCs to differentiate into cardiomyocyte-like cells [8]. It is also an anticancer drug. Salvianolic acid B (SalB) is a monomer of* Salvia miltiorrhiza* radix; recent data show that it has a benign effect on the nervous and cardiovascular systems. SalB can improve cognitive performance and has antihyperalgesic function in the treatment of neuropathic pain; it has been shown to reduce brain damage and inflammation after traumatic brain injury [9–11]. Moreover, it can protect cardiomyocytes from apoptosis, restrict the poly(ADP-ribose) polymerase-1 pathway, and guarantee the integrity of mitochondria and nuclei of cardiac tissue during acute myocardial infarction [12]. Cardiomyocyte lysis medium (CLM) is another potential source of paracrine factor for triggering the differentiation of MSCs into cardiomyocyte-like cells [13]. Our previous studies indicated that SalB can promote MSC differentiation into cardiomyocyte-like cells [14]. However, the signaling pathway through which SalB works remains unknown.

Wnt signaling is necessary for various aspects of cardiac development in embryonic stem cells, including myocardial specification, cardiac morphogenesis, and cardiac valve formation [15]. It has two branches: the beta-catenin mediates the canonical pathway and the RhoA/PLC mediates the noncanonical pathway. The noncanonical signaling pathway plays a crucial role in cardiac morphogenesis, in addition to its potential effect in cardiac specification and progenitor expansion. The canonical Wnt/beta-catenin pathway has already been reported to play a part in hematopoietic [16], mammary [17], and brain stem cell functions [18]. Experiments in mouse embryonic stem cells demonstrate that the Wnt/beta-catenin signaling pathway has a biphasic role in cardiac specification [19]: promoting cardiac gene expression early and then restraining cardiac differentiation at a later stage. The expression of Wnt/beta-catenin signaling and the activity of Wnt reporters are transiently augmented in differentiating embryo stem cells just prior to the expression of cardiac marker genes, such as GATA4 and NKx2.5. Blocking the Wnt/beta-catenin signaling pathway during the early phase of differentiation inhibits the expression of early cardiac marker genes and the emergence of beating cardiomyocytes [20]. That is, the Wnt/beta-catenin signaling pathway has a conserved role in cardiac development in vertebrates, promoting cardiac precursor cell formation and subsequently inhibiting heart muscle differentiation [21]. However, bone marrow-derived MSCs are quite different. Cho et al. reported that glycogen synthase kinase-3beta (GSK-3beta) in the cytosol induces cardiomyocyte differentiation of MSCs via downregulation of beta-catenin [22]. GSK-3beta is another pivotal component of the Wnt/beta-catenin signaling pathway. Beta-catenin is phosphorylated by GSK-3beta and then degraded by ubiquitin proteasome. Cho et al. proved that 5-aza induced cardiomyocyte differentiation by MSCs via increasing GSK-3beta expression.

5-Aza and CLM are the most frequently used conditions, but both of them have defects. To obtain a better understanding of how to gain a higher differentiation rate from MSCs to cardiomyocytes, we investigated the role of SalB combined with 5-aza and CLM. This cocktail method was tested in this experiment to investigate their roles, independent and in combination, in the differentiation of MSCs into cardiomyocyte-like cells, as well as their mechanisms. Thus, beta-catenin, GSK-3beta, and the ratio of phosphorylated/dephosphorylated GSK-3beta were detected to determine whether inhibiting the Wnt/beta-catenin signaling pathway is the key to induce MSCs differentiation into cardiomyocytes.

## 2. Materials and Methods

### 2.1. Statement of Ethics

This study involving the use of Sprague-Dawley rats was conducted in strict accordance with provisions and general recommendation of Chinese Experimental Animals Administration Legislation and was approved by the Committee on Ethics of Animal Experiments of Tianjin University of Traditional Chinese Medicine (Permit number SCXK 2005-0001). All surgeries were performed under sodium pentobarbital anesthesia, and all efforts were made to minimize suffering.

### 2.2. Materials

Low-glucose Dulbecco's modified Eagle's medium (L-DMEM), DMEM/F12, and fetal bovine serum (FBS) were purchased from Gibco (Invitrogen, Applied Biosystems, Carlsbad, CA, USA). Amphotericin, 5-azacytidine, and salvianolic acid B (SalB) were purchased from Sigma-Aldrich (St. Louis, MO, USA). D-Hank's balanced salt mixture, ABI High Capacity cDNA, Reverse Transcription kits, and Agilent Brilliant II SYBR Green qPCR Master Mix were purchased from Applied Biosystems. Rabbit anti-rat monoclonal to cTnT was purchased from Abcam (Cambridge, UK); rabbit anti-rat monoclonal to GAPDH, beta-catenin, and GSK-3beta were purchased from EPIT MICS (California, USA), and the phosphorylated GSK-3beta was purchased from CST (Boston, USA).

### 2.3. Methods

#### 2.3.1. Isolation and Culture of MSCs

MSCs were separated from the femur and the tibia marrow of male rats (180–200 g) [23]. The cells were rinsed from the marrow cavity and then centrifuged at 1000 ×g for 10 minutes. The pellets were cultured with complete medium (L-DMEM, 10% FBS, 100 U/mL penicillin, and streptomycin) at 37°C in a 5% CO_2_ atmosphere. Nonadherent cells (hematopoietic cells) were removed by replacing the medium after 2 days. The medium was changed every other day, and MSCs from the fourth (i.e., change of medium) were used for all experiments.

#### 2.3.2. Isolation of Neonatal Rat Cardiomyocytes and Preparation of CLM

The neonatal rat cardiomyocytes were isolated according to the 2008 work of Dell'Ovo et al., with some improvements [24]. The 20 neonatal rats (aged 0–2 days) were sterilized in 75% alcohol for 3 minutes after they were euthanized with CO_2_. The cardiac tissue was obtained through chest dissection; pericardium/epicardium and endocardium were discarded, and the myocardia (auricle and ventricle) were processed. We cut the cardiac tissue into 1 mm^3^ pieces, transferred them to a 50 mL sterile tube, and washed them three times with D-Hank's solution to remove excess blood. Then 5 mL of 0.0625% pancreatic enzyme solution was added to the tube, and the mixture was placed in the constant-temperature oscillating incubator at 37°C for 5 minutes at 190 rpm, and the supernatant was discarded. Then 3 mL of 0.0625% pancreatic enzyme solution and 3 mL 0.1% collagenase II solution were added to the pellets. The mixture was placed in the incubator at 37°C for 20 minutes at 190 rpm, then the supernatant was collected into a new tube, and digestion was terminated with FBS. This step was repeated three times, until almost no tissue block remained. The collected supernatant was centrifuged for 8 minutes at 1000 ×g, and the cells were cultured with complete medium (DF_12_, 20% FBS, and 1% BrdU) at 37°C in a humid 5% CO_2_ atmosphere. After 24 h, more than 90% of the cells showed spontaneous contraction under an inverted-phase contrast microscope (DMI3000B, Leica, Germany). The medium was changed every other day until the cells reached 80% confluence.

The well-conditioned cardiomyocytes were synchronized without FBS at 37°C for 12 hours under a 5% CO_2_ atmosphere. The cells were then digested with pancreatic enzymes and diluted with L-DMEM at 10^5^ cells per milliliter. The cells were frozen at −80°C for 4 hours and then unfrozen at room temperature. This step was repeated three times to ensure that the cells were fractured. Then the tube was centrifuged at 2000 ×g for 10 minutes. The supernatant was collected and filtered with 0.45 *μ*m filters and then stored at −20°C for later use.

#### 2.3.3. Identification of MSCs and Cardiomyocytes

Well-cultured MSCs were harvested by treatment with 0.25% trypsin and then incubated for 1 h at 4°C with the PE-conjugated mouse monoclonal antibodies against rat CD45 and CD34, FITC-conjugated mouse monoclonal antibodies against rat CD29 and CD90. Control tubes were incubated with FITC- and PE-conjugated antibodies against rat IgG1. The cells were washed with phosphate buffer solution (PBS) three times. Then the cells were detected by flow cytometric. The results showed that MSCs expressed CD29 and CD90, but not CD45 and CD34.

cTnT, a specific cardiomyocyte marker, was identified by immunofluorescence assay. Well-grown cardiomyocytes were washed three times with PBS and fixed for 5 minutes at room temperature in 4% paraformaldehyde. The cells were then permeabilized by incubation for 30 minutes in 0.5% Triton X-100 in PBS and blocked with 10% normal rabbit serum for 60 minutes. Then the cells were incubated with goat anti-rabbit cTnT antibody (1 : 100 diluted with PBS) at 4°C overnight. Blocked cells were incubated with rabbit anti-goat FITC-IgG antibody for 2 hours and were washed with PBS three times after each step. The cells were blocked with glycerol and were examined using a fluorescence microscope.

#### 2.3.4. Induction Method

After the fourth passage, MSCs were divided into eight groups based on different treatment conditions: (1) control group, (2) 5-aza group, (3) SalB group, (4) 5-aza+SalB group, (5) CLM group, (6) 5-aza+CLM group, (7) SalB+CLM group, and (8) 5-aza+SalB+CLM group. Each group was cultured for 2 weeks. Each group was synchronized (i.e., the medium was changed without FBS) for 24 h, induced by pharmaceuticals mentioned above for 24 h, and then substituted by complete medium (L-DMEM, 10% FBS, 100 U/mL penicillin, and streptomycin). The medium was changed every other day. The 5-aza concentration was 10 *μ*mol/L, the SalB concentration was 22 *μ*mol/L, and the ratio of CLM to complete medium was 1 : 1.

#### 2.3.5. Immunofluorescence Assay Detection of the Differentiation Rate

Induced MSCs were washed three times with PBS and fixed by incubation for 5 minutes in 4% paraformaldehyde, then permeabilized by incubation for 30 minutes in 0.5% Triton X-100 in PBS, and blocked by incubation for 60 minutes with 5% normal rabbit serum. Blocked cells were incubated for 1 hour at 37°C with goat anti-rat cTnT polyclonal antibody (dilution 1 : 100) at 4°C overnight. The secondary antibody, rabbit anti-goat FITC-IgG (dilution 1 : 100), was added to the cells, which were then incubated for 30 minutes at room temperature. Nuclei were stained by incubation with 4,6-diamidine-2′-phenylindole (DAPI) for 10 minutes at room temperature. The cells were washed with PBS three times after each step, blocked with glycerol, and examined under a fluorescence microscope.

#### 2.3.6. Quantitative Real-Time PCR Detection of the mRNA Level

Total RNA was extracted from cultured MSCs, which had been induced for 2 weeks, using an RNA kit (Sigma-Aldrich, E.N.Z.A. DNA/RNA/protein isolation kit) according to the manufacturer's instructions. The RNA concentration was determined using a microspectrophotometer device. The primer sequences are shown in Table 1. We synthesized cDNA from 2 *μ*g of total RNA, according to the manufacturer's instructions. The quantitative reaction was conducted according to the qPCR kit. The reference gene was GAPDH, and its threshold cycles were 22.

#### 2.3.7. Western Blotting Detection of the Protein Expression

Proteins were obtained from adherent cells. Quantification of the protein was conducted using a modified bicinchoninic acid (BCA) assay (Cwbio, China). Protein samples were prepared by boiling them for 3 minutes after the addition of a loading buffer (Cwbio, China). Proteins were then separated using sodium dodecyl sulfate polyacrylamide gel electrophoresis (SDS-PAGE) on 8% or 10% gel and transferred onto polyvinylidene fluoride (PVDF) membrane by electroblotting. After being blocked in nonfat milk for 1 h, the membranes were incubated at 4°C overnight with the primary antibody. The following day, the membranes were washed and incubated with the goat anti-rabbit horseradish peroxidase-conjugated secondary antibody at room temperature for 1 h. Membranes were washed, and protein bands were visualized using an enhanced chemiluminescence kit (Cwbio, China) under a ChemiDoc imaging system (Bio Rad Laboratories, Berkeley, CA, USA).

### 2.4. Statistical Analysis

Each experiment was conducted three times. Statistical analyses were performed using one-way analysis of variance (ANOVA). All data are expressed as mean ± SD. Significant difference was considered *P* < 0.05.

## 3. Results

### 3.1. A Method for Purification of MSCs and Cardiomyocytes Was Established

**Figure 1:**
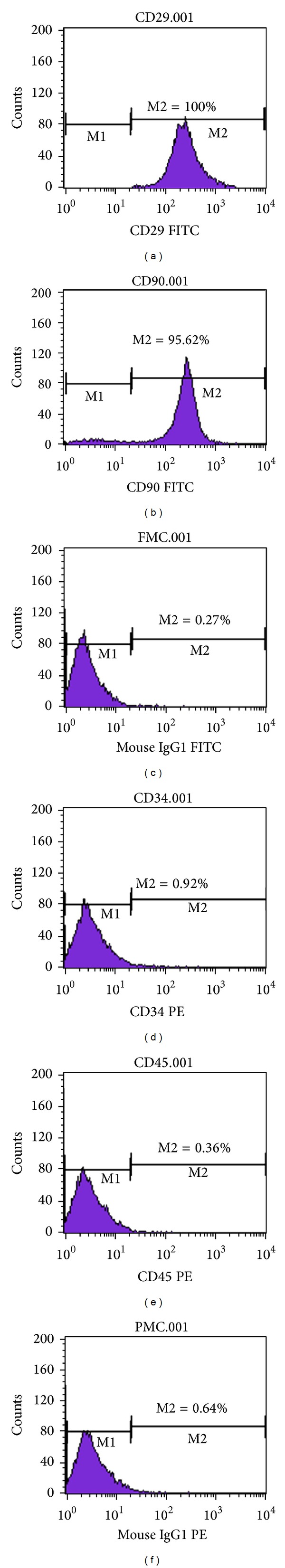
Identification of MSCs. The purified MSCs were identified by flow cytometry. MSCs were derived from the bone marrow of adult SD rats. (a) CD29 positive percent is 100%, (b) CD90 positive percent is 95.62%, (d) CD34 positive percent is 0.92%, and (e) CD45 positive percent is 0.64%.

**Figure 2:**
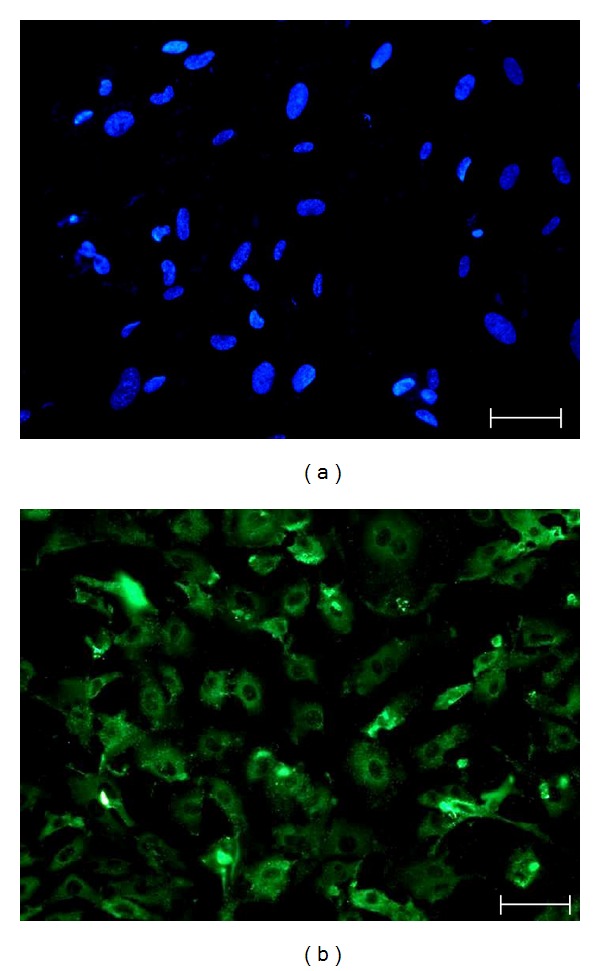
(a) Negative control for staining IgG in MSCs. The negative control for detecting cTnT expression in MSCs after induction for two weeks. Scale bar = 50 *μ*m. (b) Identification of cardiomyocytes. Purified cardiomyocytes derived from new-born rats' heart tissue were identified by immunoflourescence assay. Immunofluorescence staining of cTnT in cardiomyocytes was positive. Scale bar = 10 *μ*m.

Cultured MSCs were identified by a flow cytometer (BD USA). MSCs were positive for CD90 and CD29, but negative for CD45 and CD34 (Figure 1). The cardiomyocyte-specific marker cTnT was identified by immunofluorescence assay. The cultured cardiomyocytes were positive for cTnT (Figure 2(b)).

### 3.2. SalB Combined with 5-Aza and CLM Improved Cardiomyocytes Differentiation Rate from MSCs

**Figure 3:**
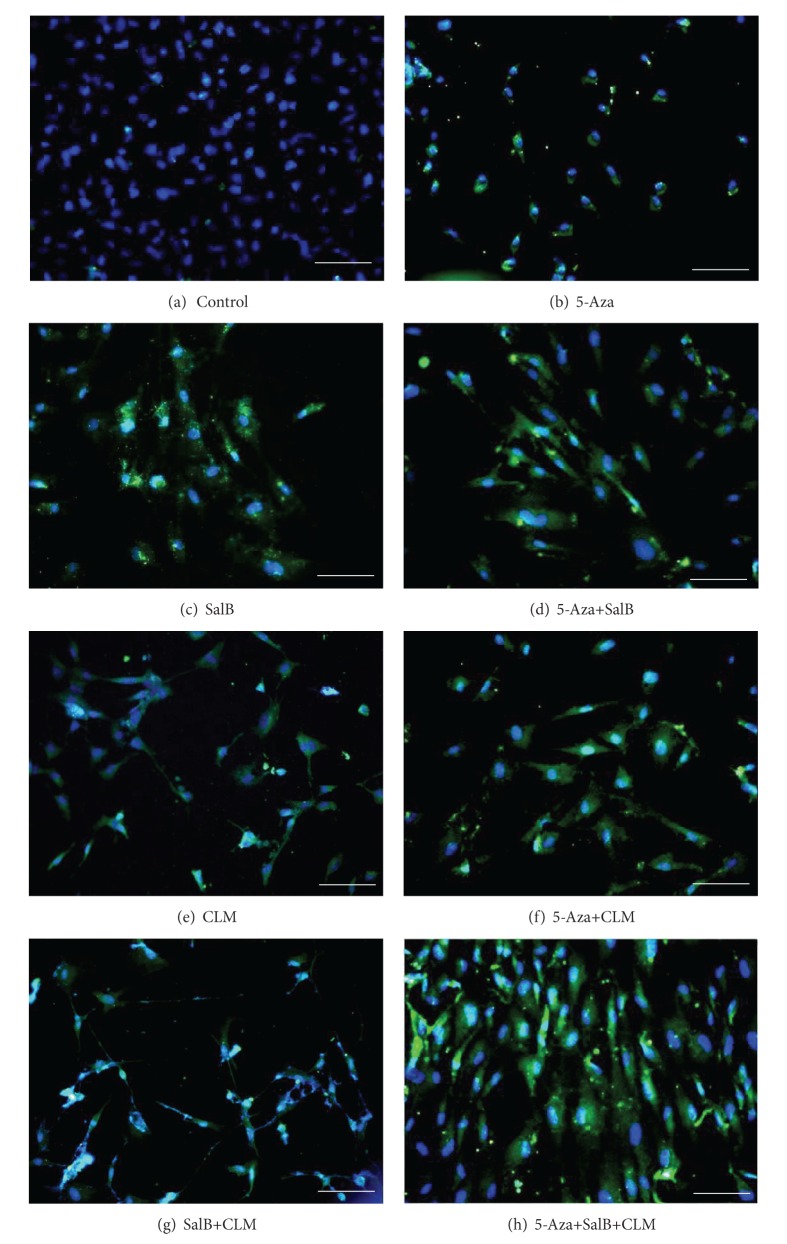
Immunofluorescence staining of cTnT in MSCs. After induction for two weeks, MSCs in each group were identified by immunofluorescence staining of cTnT. (a) Control group; (b) 5-aza group; (c) SalB group; (d) 5-aza+SalB group. (e) CLM group; (f) 5-aza+CLM group; (g) SalB+CLM group; (h) 5-aza+SalB+CLM group. Scale bar = 50 *μ*m.

After the MSCs were induced for 2 weeks under different conditions, the differentiation rate was detected by immunofluorescence assay (Figure 3). There was no cTnT expression in the control group. cTnT expressions in each induced group were very different, the 5-aza+salB group was much higher than the 5-aza group and salB group. The 5-aza+salB+CLM group was much higher than the 5-aza+CLM group and salB+CLM group.

**Figure 4:**
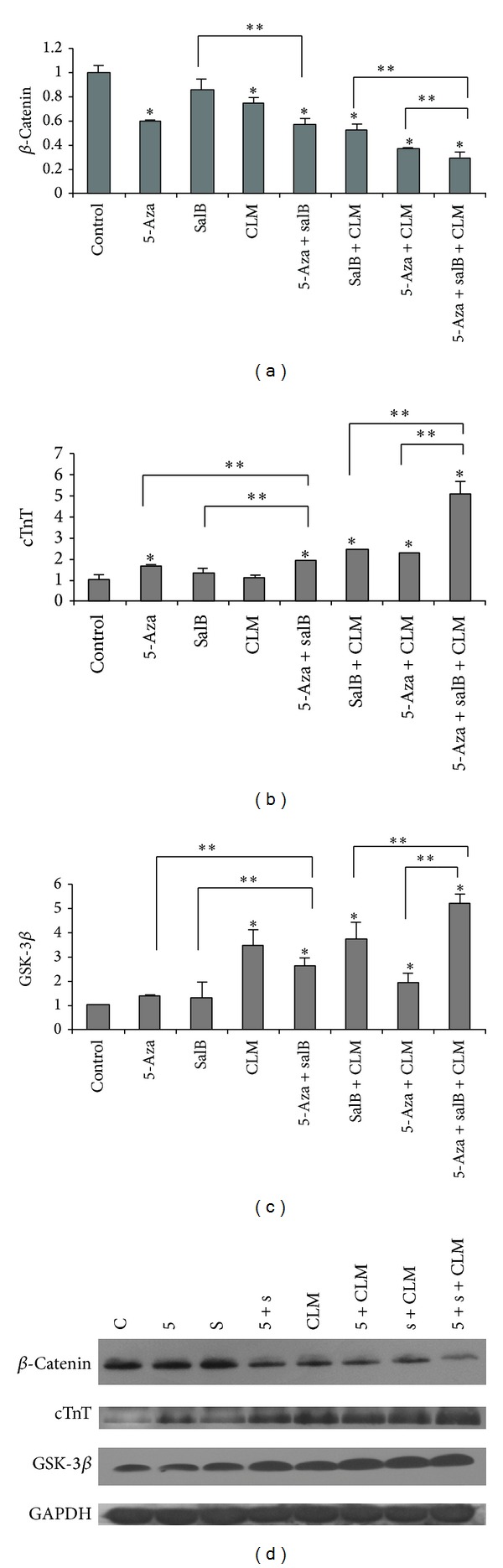
mRNA and protein expressions of beta-catenin, cTnT, and GSK-3beta detected by qPCR and Western blotting in MSCs. (a) Beta-catenin mRNA expression; (b) cTnT mRNA expression; (c) GSK-3beta mRNA expression; (d) protein expression of beta-catenin, cTnT, and GSK-3beta was detected by Western blotting. The group sequence from left to right is control group, 5-aza, SalB, 5-aza+SalB, CLM, 5-aza+CLM, SalB+CLM, and 5-aza+SalB+CLM. Results are shown as the mean ± SD values. Experiments were repeated three times. *P < 0.05 compared to the control group. **P < 0.05 compared between the two groups as shown in the figure.

**Figure 5:**
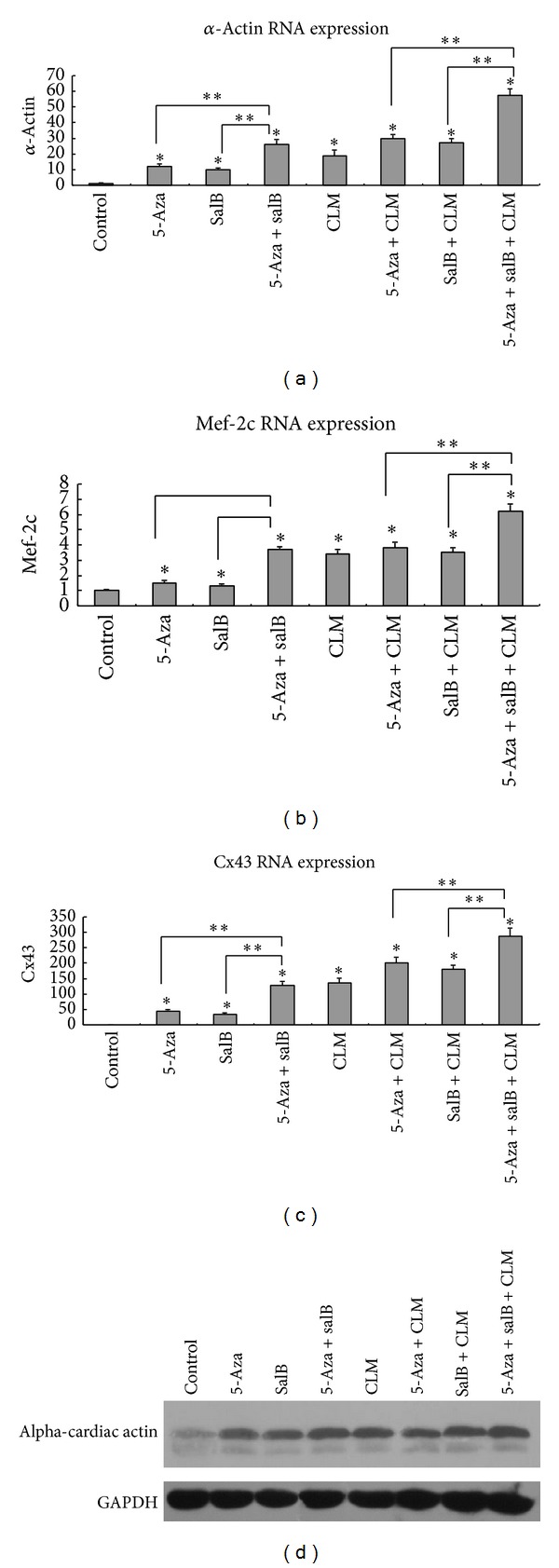
mRNA expression of alpha-cardiac actin, mef-2c, and cx43 detected by qPCR. The reference gene is GAPDH, and its threshold cycles were 22. (a) Alfa-actin mRNA expression; (b) mef-2c mRNA expression; (c) cx43 mRNA expression. (d) Alpha-cardiac actin protein expression. Results are shown as the mean ± SD values. Experiments were repeated three times. *P < 0.05 compared to the control group. **P < 0.05 compared between the two groups as shown in the figure.

**Figure 6:**
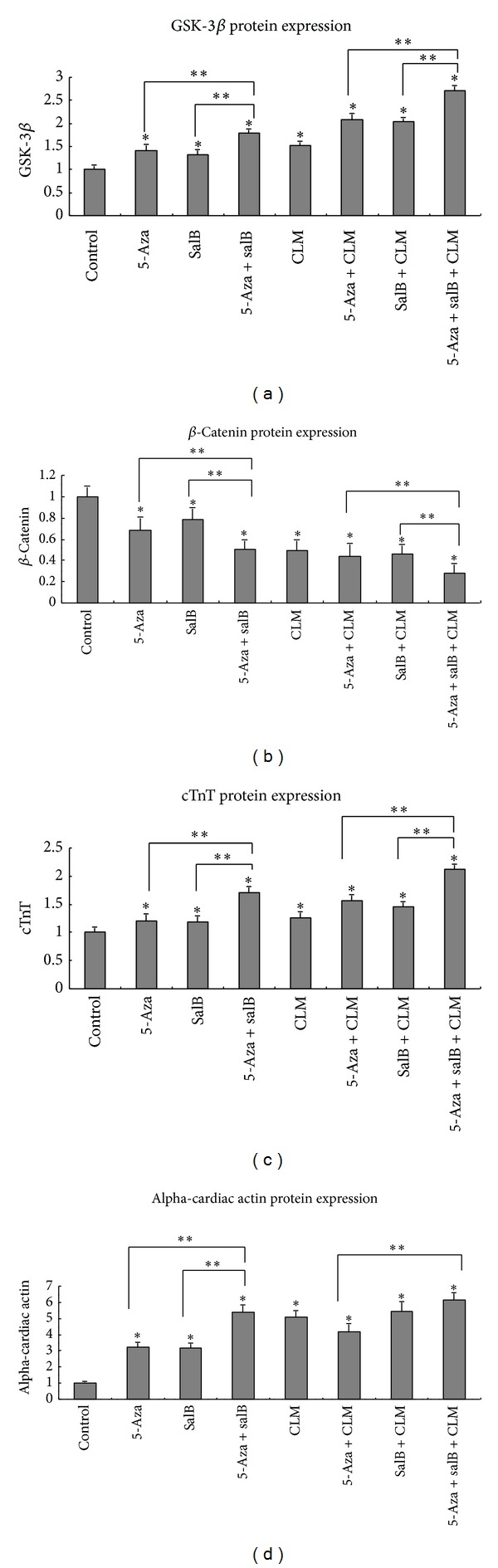
Relative protein expression. Expression of GSK-3beta, beta-catenin, cTnT, and alpha-cardiac actin was quantified by densitometric analysis of the immunoblots. *P < 0.05 compared to the control group. **P < 0.05 compared between the two groups as shown in the figure.

For further analysis of cardiomyocyte differentiation, quantitative real-time PCR and Western blotting were conducted to detect the mRNA and proteins of cTnT, alpha-cardiac actin, beta-catenin, and GSK-3beta. Compared to the control group, the expression of beta-catenin mRNA declined (Figure 4(a)), while the expression of cTnT, alpha-cardiac actin, and GSK-3beta mRNA increased in the inducing groups (Figures 4(b), 4(c), and 5(a)). In addition, the expression of other cardiomyocytes-related markers such as mef-2c and cx43 increased considerably compared to the control group (Figures 5(b) and 5(c)). Protein expression was similar to that of the mRNA (Figures 4(d), 5(d), and 6). As cTnT and alpha-cardiac actin expression increased, GSK-3beta expression increased, and beta-catenin expression decreased, particularly in the 5-aza+salB and 5-aza+SalB+CLM group.

### 3.3. MSCs Differentiate into Cardiomyocytes through Inhibiting the Wnt/Beta-Catenin Signaling Pathway

**Figure 7:**
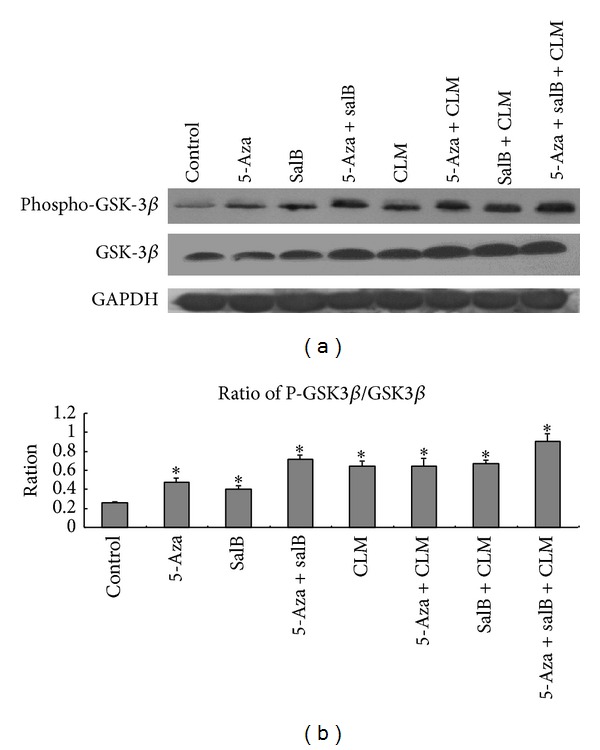
Phosphorylated GSK-3beta protein expression. (a) Phospho-GSK-3beta protein expression by Western blotting. (b) Phospho-GSK-3beta was quantified by densitometric analysis of the immunoblots. The group sequence from left to right is control group, 5-aza, SalB, 5-aza+SalB, CLM 5-aza+CLM, SalB+CLM, and 5-aza+SalB+CLM. *P < 0.05 compared to the control group.

The ratio of phosphorylated/dephosphorylated GSK-3beta increased in the tested groups (Figure 7), which means that phospho-GSK-3beta plays important role in MSCs differentiation into cardiomyocytes. The 5-aza+salB group was almost 1.5-fold the 5-aza group and salB group. The 5-aza+salB+CLM group was almost 1.4-fold the 5-aza+CLM group and salB+CLM group. The phospho-GSK-3beta directly degraded beta-catenin, leading to loss of function in the Wnt/beta-catenin signaling.

## 4. Discussion

All of the MSCs used in this study were chosen from the fourth passage, as described. MSCs can undergo spontaneous transformation and lose their potential to differentiate into cardiomyocyte-like cells after long-term culture* in vitro*. Zhang et al. investigated the passage-restricted differentiation potential of MSCs into cardiomyocytes and its relationship to proliferation capacity [25]. Their findings suggest that the differentiation potential of MSCs into cardiomyocytes depends on proliferation ability and that differentiation occurs in passage-restricted patterns. The arrest of cell growth is one of the preconditions for inducing the expression of cardiomyocyte-specific genes. In contrast to the rapid proliferation rate in the first- and eighth-passage MSCs, the fourth-passage MSCs entered a growth-arrested phase. A similar growth pattern in MSCs was demonstrated by Liu et al. [26]. They discovered the morphological transformation and the expression of the cardiomyocyte markers in fourth-passage MSCs, but not in other following successive passages under the same culture and induction conditions. That is to say, fourth-passage MSCs, rather than first-passage or eighth-passage MSCs, can differentiate into cardiomyocyte-like cells. The differentiation ability of MSCs into cardiomyocyte-like cells is limited by proliferation capacity, and the cell growth arrest does not imply the loss of the differentiation potential of MSCs.

In the present study, we demonstrated that salB combined with 5-aza and CLM can get a higher differentiation rate of MSCs into cardiomyocyte-like cells. The RNA expression of cardiomyocyte-related markers such as cTnT, alpha-cardiac actin, mef-2c, and Cx43 in the 5-aza+salB group is much higher than the 5-aza group and salB group; the same occurs in the 5-aza+salB+CLM group, the 5-aza+CLM group, and the salB+CLM group. The protein expression of cTnT in the 5-aza+salB group is almost 1.5-fold that of the 5-aza group and salB group. The protein expression of cTnT in the 5-aza+salB+CLM group is almost 1.6-fold that of the 5-aza+CLM group and the salB+CLM group. Therefore, we can conclude that SalB combined with 5-aza and CLM can improve the cardiomyocyte differentiation rate from MSCs. SalB has a cardioprotective function at certain levels through multiple targets related to NO production [27], and SalB can also prevent ischemia-reperfusion injury by inhibiting DNA synthesis of noncardiomyocyte and stress-activated protein (SAP) kinase activity [28]. CLM provides an appropriate microenvironment for cardiomyocytes and cytokines in the culture medium. However, it is not so effective when applied alone, which is consistent with the report that direct cell-to-cell contact between MSCs and cardiomyocytes is needed for the differentiation of MSCs into cardiomyocytes [3].

Our results suggest that inhibition of the Wnt/beta-catenin signaling pathway is part of the mechanism of MSC differentiation into cardiomyocytes. Compared to the control group, after the MSCs were induced by 5-aza+SalB+CLM, the cTnT expression was 5.1-fold higher and the GSK-3beta expression was 5.2-fold higher, while the beta-catenin expression was 0.4-fold higher. The ratio of phosphorylated/dephosphorylated GSK-3beta in the tested groups was significantly higher than in the control group. The 5-aza+salB group was significantly higher than in the 5-aza group and salB group, and the 5-aza+salB+CLM group was significantly higher than in the 5-aza+CLM group and salB+CLM group. Since the phosphorylated GSK-3beta leads to degradation of beta-catenin directly, we can conclude that the higher phosphor-GSK-3beta expression blocked the Wnt/beta-catenin signaling pathway.

Upon binding of Wnts (Wnt1, 3*α*, and 8) to their receptors such as Frizzled and low-density lipoprotein receptor-related protein 5/6 (LRP5 and LRP6), the Wnt/beta-catenin signaling pathway is then activated. The signal is then transduced through the disheveled protein to negatively regulate glycogen synthase kinase-3beta (GSK-3beta), leading to the accumulation of beta-catenin in the cytoplasm. Beta-catenin is then transported into the nucleus, where it forms a complex with the T-cell factor/lymphocyte enhancer factor (TCF/LEF) family and activates the expression of genes such as* c-myc* or* cyclin D1*. In the absence of Wnt signals, cytoplasmic beta-catenin forms a complex with scaffolding proteins APC and axin and is phosphorylated at specific serine and/or threonine residues by GSK-3beta. These phospho-Ser/Thr residues are recognized by an ubiquitin ligase complex, which results in the degradation of beta-catenin. Thus, GSK-3beta plays a key role in the regulation mechanism for the Wnt/beta-catenin pathway [29]. GSK-3beta regulates many major signaling proteins involving in cell growth and differentiation, such as Wnt, Notch, and hedgehog.

Many studies have provided evidence that blocking the Wnt/beta-catenin pathway in any position can promote MSCs to differentiate into cardiomyocyte-like cells. Cho et al. showed that cardiac dysfunction can be attenuated after myocardial infarction through injection with MSCs that overexpress GSK-3beta. In an* in vitro* study, we showed that overexpression of GSK-3beta in MSCs promotes the expression of cardiomyocyte marker genes and proteins; at the same time, it prevents the expression of noncardiac markers, such as neuronal markers [30]. sFRP2, being released from bone marrow-derived mononuclear cells, is an inhibitor of canonical Wnt signaling. It plays a major role in regulating the paracrine effects of MSCs on cardiac tissue repair [31]. Intramyocardial injection of MSCs that overexpress sFRP2 leads to strengthened engraftment and vascular density, decreased infarct size, and enhanced cardiac function after myocardial infarction in mice. Thus, sFRP2 is an important molecule for the biogenesis of a new regenerative phenotype character in MSCs, and Wnt signaling is a therapeutic target for heart diseases [32]. It is sufficient to generate cardiomyocytes from human embryonic stem cells by block Wnt signaling through pharmacological or other methods. Furthermore, suppression of Wnt signaling does not generate other mesoderm lineages [33]. Egea et al. found that activation of the Wnt/beta-catenin signaling pathway by knockdown of the tissue inhibitor of metalloproteinase-1 (TIMP-1) promotes osteogenic differentiation by human MSCs [34].

Li et al. proved that the differentiation of MSCs into cardiomyocytes is dependent on the Notch signal. Jagged 1 protein can activate Notch signaling and increase the differentiation rate of MSCs into cardiomyocytes, while its effect can be restrained by DAPI [35]. Transduction of Wnt 11, which activates the noncanonical Wnt signaling into MSCs, enhances MSC differentiation into cardiomyocytes through upregulating GATA-4 [36]. GATA-4, a cardiac transcription factor, plays a key role in cell-lineage differentiation during development. GATA-4 overexpression in MSCs significantly increases differentiation rate into a myocardiocyte phenotype, which may be related to the upregulation of the insulin-like growth factor-binding protein-4. This body of evidence proves that it is necessary to inhibit the Wnt/beta-catenin signaling pathway when inducing MSCs to differentiate into cardiomyocytes.

According to molecular analysis, treated cells acquired the molecular phenotype of cardiomyogenic cells; although qPCR and Western blotting showed significant differences in mRNA expression and protein synthesis, the cardiomyocyte-like cells did not beat. These results strongly suggest that the soluble factors alone were not sufficient to induce differentiation of MSCs and that physical contact between cardiomyocytes and MSCs is a requisite. The factors of microenvironment involved cell-to-cell interaction; cytokines and extracellular matrix protein are needed to induce differentiation. More experiments were needed to achieve a big step in MSC differentiation. In spite of the efforts to enhance the differentiation of MSCs into cardiomyocytes, less than 30% of MSCs contribute to cardiomyocytes differentiation; the others contribute to osteogenesis, chondrogenesis, adipogenesis, or other processes [25]. Single-cell monoclonal technology can preliminarily purify this subpopulation of MSCs with a high potential for cardiomyocyte differentiation, but the specific surface markers for these cells remain unknown.

In conclusion, 5-aza, salB, and CLM are three individual induction agents for the differentiation of MSCs into cardiomyocyte-like cells. They have additive effects in this process when used in combination. The mechanisms of action of this induction may involve inhibition of the canonical Wnt signaling pathway. The differentiation of MSCs into cardiomyocytes has many induction requirements: pharmaceutical induction, cytokines, microenvironment, cell-to-cell contact, ion concentration, and so on. It is clear that inhibiting the canonical Wnt signaling is necessary for MSCs to differentiate into cardiomyocytes, but whether the activation of noncanonical Wnt signaling or Notch signaling combined with pharmaceutical treatment can induce MSCs to differentiate into beating cardiomyocytes requires further exploration.

## Figures and Tables

**Table 1:** Primers for real-time quantitative PCR.

Gene	Upstream	Downstream
GAPDH	GTCTTCTGAGTGGCAGTGAT	TGCTGAGTATGTCGTGGAG
cTnT	GCAGGCTCTTCATGCCCAACT	CGCTCTGCCCGACGCTTTT
Beta-catenin	TCCACCAGTTGCCTTTATCA	CTGAGAAACTTGTCCGATGC
GSK-3beta	GTACCAGAACAGCCAGGGCTAC	CGGTCGGTTACTAGCAGAGCTT
Alpha-actin	TCTATGAGGGCTACGCTTTG	GCCAATAGTGATGACTTGGC
Mef-2c	GCAGACGATTCAGTAGGT	CCAGTGGCAGAAGATTAG
Cx43	GAAGGATCC**ATG**AGCGATCCTTACCACGCC	GCTTGAATTCCAAGCCGGTTTAAATCTCC
